# A Combination of Leucine, Metformin, and Sildenafil Treats Nonalcoholic Fatty Liver Disease and Steatohepatitis in Mice

**DOI:** 10.1155/2016/9185987

**Published:** 2016-11-30

**Authors:** Antje Bruckbauer, Jheelam Banerjee, Lizhi Fu, Fenfen Li, Qiang Cao, Xin Cui, Rui Wu, Hang Shi, Bingzhong Xue, Michael B. Zemel

**Affiliations:** ^1^NuSirt Biopharma Inc., 11020 Solway School Rd, Knoxville, TN 37931, USA; ^2^Center for Obesity Reversal, Department of Biology, Georgia State University, 33 Gilmer Street SE, Atlanta, GA 30302, USA

## Abstract

Sirt1, AMPK, and eNOS modulate hepatic energy metabolism and inflammation and are key players in the development of NASH. L-leucine, an allosteric Sirt1 activator, synergizes with low doses of metformin or sildenafil on the AMPK-eNOS-Sirt1 pathway to reverse mild NAFLD in preclinical mouse models. Here we tested a possible multicomponent synergy to yield greater therapeutic efficacy in NAFLD/NASH. Liver cells and macrophages or an atherogenic diet induced NASH mouse model was treated with two-way and three-way combinations. The three-way combination Sild-Met-Leu increased hepatic fatty acid oxidation and reduced lipogenic gene expression and inflammatory marker* in vitro*. In mice, Sild-Met-Leu reduced the diet induced increases of ALT, TGF*β*, PAI-1, IL1*β*, and TNF*α*, hepatic collagen expression, and nearly completely reversed hepatocyte ballooning and triglyceride accumulation, while all two-way combinations had only modest effects. Therefore, these data provide preclinical evidence for therapeutic efficacy of Sild-Met-Leu in the treatment of NAFLD and NASH.

## 1. Introduction

Nonalcoholic steatohepatitis (NASH), the progressive form of nonalcoholic fatty liver disease (NAFLD), is characterized by the presence of >5% macrovesicular steatosis, inflammation, and liver cell ballooning [1]. Its prevalence is increasing concomitantly with prevalence of obesity and diabetes, thus representing a serious public health issue [2, 3]. About 30 to 40% of NASH progresses to fibrosis or to cirrhosis, resulting in a high risk for cardiovascular and liver-related morbidity and mortality [3]. However, treatment is presently limited to lifestyle intervention, as approved treatment options are lacking and represent a significant unmet need.

Sirt1 enzyme and AMPK are important regulators of energy metabolism and modulate hepatic glucose and lipid metabolism. In addition, Sirt1 regulates multiple inflammatory pathways such as NF-*κ*B and TNF*α* [2]. Thus they play an important role in the pathophysiology of NAFLD and NASH [2, 4, 5]. Liver-specific deletion of Sirt1 results in hepatic steatosis and inflammation in mice [6], while treatment with Sirt1 activators or Sirt1 overexpression ameliorates fatty liver and reduces lipogenic gene expression [5, 7].

We have previously demonstrated that leucine acts as a direct Sirt1 activator by lowering the activation energy for NAD^+^ and enables coactivation with other AMPK/Sirt1 activators thereby reducing the necessary concentration for each individual compound [8, 9]. Synergy with leucine was also demonstrated with metformin (met), the first-line treatment drug for diabetes, at which effects are also mediated by merging on the AMPK/Sirt1 pathway [10, 11]. Accordingly, treatment with a Met-Leu combination resulted in reduction of lipid accumulation* in vitro* and reversal of hepatic steatosis* in vivo* in a HFD-induced NAFLD mouse model [12].

The endothelial nitric oxide synthase, nitric oxide and cyclic guanosine monophosphate (eNOS-NO-cGMP) signaling pathway has also been shown to affect the progression of NAFLD to NASH. High-fat diet feeding reduced eNOS-NO signaling in the liver of NAFLD models of mice and rats. This was precedent to the onset of hepatic inflammation and insulin resistance and was prevented by daily administration of sildenafil [13, 14].

The primary action of sildenafil is the inhibition of phosphodiesterase 5 (PDE5) which hydrolyses cGMP and thus terminates cGMP signaling. In addition, sildenafil activates eNOS resulting in increased NO/cGMP signaling with consecutive activation of the cGMP-dependent protein kinases (PKGs) to induce vasodilatory, anti-inflammatory, and antiproliferative effects [15–18].

This pathway also interacts with the sirtuin pathway, as it stimulates Sirt1, while Sirt1 appears to deacetylate and activate eNOS and thereby elevate NO levels; thus sildenafil's effects may be partly mediated by Sirt1 activation [17, 19–21]. Moreover, leucine synergizes with PDE5 inhibitors to exert amplifying downstream effects of AMPK and Sirt1 activation on glucose and fat metabolism as well as reversal of hepatic steatosis and inflammation* in vitro* and* in vivo* [22]. Accordingly, the aim of this study was to evaluate the effects of a three-way interaction between leucine, metformin, and sildenafil on AMPK/Sirt1/eNOS pathway and the protective effects on hepatocyte metabolism in a NASH mouse model.

## 2. Methods

### 2.1. Cell Culture

Human hepatoma HepG2 cells (ATCC, Manassas, VA, USA) were grown in Dulbecco's modified Eagle's medium (DMEM, 5.5 mM glucose) containing 10% fetal bovine serum (FBS) and antibiotics (1% penicillin-streptomycin) at 37°C in 5% CO_2_ in air. Mouse AML-12 liver cells (ATCC, Manassas, VA, USA) were grown and maintained in 1 : 1 mixture of DMEM and Ham's F12 medium with 0.005 mg/mL insulin, 0.005 mg/mL transferrin, 5 ng/mL selenium, 40 ng·mL dexamethasone, 10% FBS, and antibiotics (1% penicillin-streptomycin) at 37°C in 5% CO_2_ in air. Mouse RAW 264.7 macrophages (ATCC, Manassas, VA, USA) were grown and maintained in DMEM containing 10% fetal bovine serum (FBS) and antibiotics (1% penicillin-streptomycin) at 37°C in 5% CO_2_ in air. Media were replaced with fresh medium every 2 to 3 days. Cells were split at a 1 : 4 ratio at 70 to 80% confluence.

Lipid accumulation in HepG2 cells was induced by incubation in 25 mM glucose DMEM media for 48 hours. Lipid accumulation and inflammatory response in AML-12 cells and RAW 264.7 macrophages were induced by stimulation with 500 *μ*M free fatty acids (FFA, palmitic-oleic acid mixture 1 : 2) and lipopolysaccharide (LPS, 1 ng/mL) for 24 hours.

Treatment (metformin 0.1 mM, leucine 0.5 mM, and sildenafil 1 nM) was added for further 24 to 48 hours.

### 2.2. Coculture

Mouse AML-12 liver cells and RAW 264.7 macrophages were seeded together in a ratio 4 : 1. Next day lipid accumulation and inflammatory response were induced by stimulation with 500 *μ*M free fatty acids (palmitic-oleic acid mixture 1 : 2) and LPS (1 ng/mL) for 24 hours. The cells were then treated as indicated for 24 hours.

### 2.3. MCP1 and TNF*α* Measurement in Media

AML-12 and/or RAW 264.7 macrophages were seeded and treated as described above. At the end of the treatment, the media were harvested. Monocyte chemotactic protein- (MCP-) 1 and tumor necrosis factor- (TNF-) *α* secretion was measured with the MCP1 Mouse Elisa kit and TNF-alpha Mouse Elisa kit (Abcam, Cambridge, MA, USA), respectively, according to manufacturer's instructions.

### 2.4. Western Blot

The Sirt1, phospho-AMPK (Thr172), AMPK, FAS, SCD1, PPAR-*α*, and PPAR*δ*, SREBP1, and TNF-*α* antibodies were obtained from Cell Signaling (Danvers, MA). Protein levels of cell extracts were measured by bicinchoninic acid assay (BCA) kit (Thermo Fisher Scientific Inc., Waltham, MA). For Western blot, 10–50 *μ*g protein was resolved on 4–15% gradient polyacrylamide gels (criterion precast gel, Bio-Rad Laboratories, Hercules, CA), transferred to PVDF or nitrocellulose membranes, incubated in blocking buffer (5% nonfat dry milk in TBS), and then incubated with primary antibody (1 : 1000 dilution), washed, and incubated with horseradish peroxidase- or fluorescence-conjugated secondary antibody (1 : 10000 dilution). Visualization was conducted using Li-COR Odyssey Fc Imaging system (Li-COR Biosciences, Lincoln, NB) and band intensity was assessed using Quantity One (Bio-Rad Laboratories, Hercules, CA), with correction for background and loading controls.

### 2.5. Fatty Acid Oxidation

Cellular oxygen consumption was measured using a Seahorse Bioscience XF24 analyzer (Seahorse Bioscience, Billerica, MA) in 24-well plates at 37°C. HepG2 cells were seeded at 40,000 cells per well. Lipid accumulation was induced by 48 h incubation with 25 mM glucose. Cells were treated for 24 hours with the indicated treatments, washed twice with nonbuffered carbonate-free pH 7.4 low glucose (2.5 mM) DMEM containing carnitine (0.5 mM), equilibrated with 550 *μ*L of the same media in a non-CO_2_ incubator for 30 minutes, and then inserted into the instrument for 15 minutes of further equilibration. O_2_ consumption was measured in three successive baseline measurements at eight-minute intervals prior to injection of palmitate (200 *μ*M final concentration). Post-palmitate-injection measurements were taken over a 3-hour period with cycles consisting of 10 min break and three successive measurements of O_2_ consumption. The area under the curve was calculated.

### 2.6. Animals and Diets

Six- to eight-week-old male C57BL/6J mice were purchased from Jackson Laboratories. First NASH was induced in all animals (except low-fat diet control animals (LF)) via feeding of a high-fat atherogenic diet (HC: 60% of calories from fat, 1.25% cholesterol, and 0.5% cholate) for 6 weeks. After this induction period, the HC animals were randomized into one of the following groups with 10 animals/group and kept on their experimental diet for additional 6 weeks (12 weeks total): high-fat atherogenic diet (HC); HC + sildenafil (25 mg/kg diet, calculated as human equivalent dose of 1 mg/day) (HC + Sil); HC + leucine (24 g/kg diet) + sildenafil (HC + Leu + Sil); HC + leucine + metformin (0.25 g/kg diet, calculated as a human equivalent dose of 250 mg/day (HC + Leu + Met)); HC + metformin + sildenafil (HC + Met + Sil); HC + leucine + metformin + sildenafil (HC + Leu + Met + Sil). The LF animals were continued on their diet for an additional 6 weeks.

Animals were housed in polypropylene cages at a room temperature of 22°C and a 12 h light/dark cycle. The animals had free access to water and their experimental food throughout the experiment. Body weight was measured every week. At the end of the treatment period (6 weeks) all animals were humanely euthanized with CO_2_ inhalation. Blood was collected via trunk bleed and tissues were collected for further experiments as described below.

This study and all animal procedures were performed under the auspices of Institutional Animal Care and Use Committee-Approved protocol of the Georgia State University and in accordance with PHS policy and recommendations of the Guide.

### 2.7. Liver Histology

Liver tissues were fixed in 10% neutral formalin, embedded in paraffin, and cut into 5 *µ*m sections. Sections were processed for hematoxylin and eosin (H&E) staining and histological images were recorded using Nikon Eclipse E800 Microscopy with Zeiss AxioCam camera.

### 2.8. Liver Triglyceride Measurements

Liver lipid extraction was conducted as previously described with minor modifications [23]. Briefly, ~100 mg of liver was thawed, minced, and weighted in glass tube. Lipids were extracted in 2 : 1 CHCl_3_/methanol at room temperature overnight. The lipid portion was then dried down under N_2_ and redissolved in a measured volume of 2 : 1 CHCl_3_/methanol. Diluted H2SO4 was added to the sample, which was then vortexed and centrifuged to split the phases. The aqueous upper phase was aspirated and discarded, and an aliquot of the bottom phase was dried down and dissolved in 2% Triton X-100. The triglyceride content was then measured using TG kit/L-Type TG M (Wako Chemicals, USA) and normalized to liver weight.

### 2.9. ALT Measurement

Serum ALT levels were measured in fed mice after 4 weeks of diet treatment using a mouse ALT ELISA kit from BioVision.

### 2.10. Liver CD68 and Collagen Staining

Liver tissues were fixed in 10% neutral formalin, embedded in paraffin, and cut into 5 *µ*m sections. For inflammation immunostaining, slides were immunoblotted with CD68 (Bio-Rad MCA 1957) as primary antibody and Biotin-SP-AffiniPure Mouse Anti-Rat IgG as secondary antibody. This was followed by the application of the immunoperoxidase technique with a Vector kit. Areas of staining were quantified with ImageJ and expressed as percentages of the field area. For fibrosis Picro Sirius Red staining, liver slides were dewaxed and hydrated, Weigert's hematoxylin stained for 8 minutes, and Picro-Sirius Red (Picro Sirius Red Stain Kit, Abcam, Cat # ab150681) stained for one hour. Acidified water wash was applied. Slides were dehydrated in three changes of 100% ethanol and cleared in xylene and mounted in a resinous medium. All of the histological images were recorded using Nikon Eclipse E800 Microscopy with Zeiss AxioCam camera. Areas of staining were quantified with ImageJ and expressed as percentages of the field area.

### 2.11. Gene Expression

#### 2.11.1. *In Vitro* Data

Cells were grown in a 96-well plate. Cell Lysis, reverse transcription, and RT-PCR were performed using the TaqMan® Gene Expression Cells-to C_T_™ Kit (Life Technologies, Cat # 4399002) according to manufacturer's instructions. Gene expression was assessed by RT-PCR using StepOnePlus™ PCR system (Thermo Fisher Scientific) and TaqMan Gene expression assays for AMPK (Life Technologies, Cat # Mm01264789) and Sirt1 (Life Technologies, Cat # Mm01168521).

#### 2.11.2. *In Vivo* Data

Total RNA from liver was extracted using the Tri-Reagent kit (Molecular Research Center, Cincinnati, OH) and gene expression was assessed by quantitative reverse transcription- (RT-) PCR (ABI Universal PCR Master Mix, Applied Biosystems, Foster City, CA) using a Stratagene Mx3000p thermocycler (Stratagene, La Jolla, CA). Cyclophilin was used to normalize the gene expression data. The primer and probe sets used in the assays were purchased from Applied Biosystems/Life Technologies (Grand Island, NY).

### 2.12. Statistical Analysis

All data are expressed as mean ± SEM. Data were analyzed by one-way ANOVA, and significantly different group means (*P* < 0.05) were separated by the least significant difference test using GraphPad Prism version 6 (GraphPad Software, La Jolla, California, USA, www.graphpad.com).

## 3. Results

**Figure 1:**
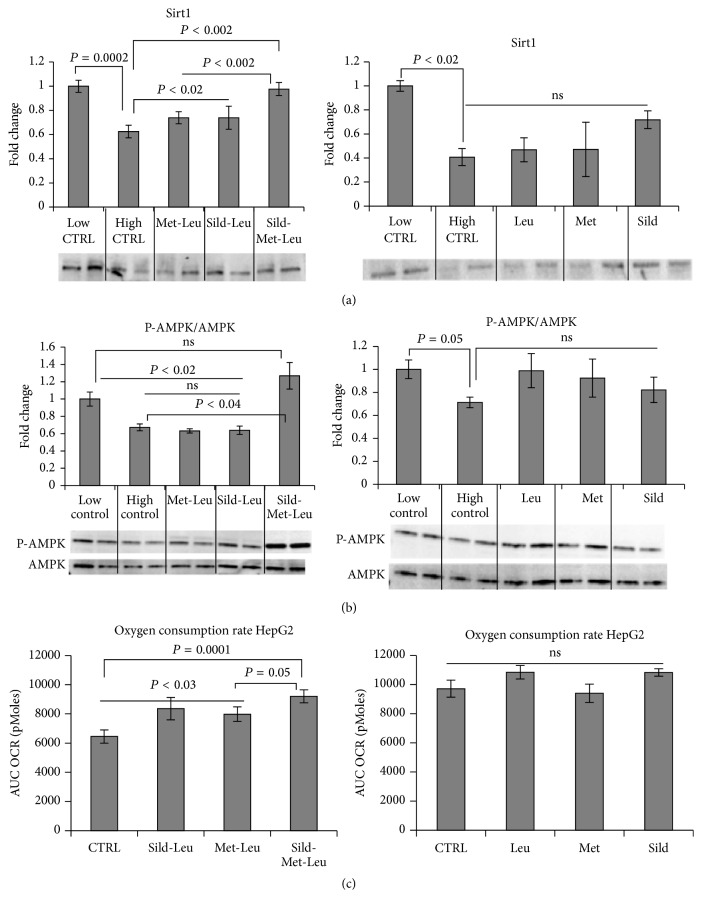
Sild-Met-Leu treatment in hepatocytes increases AMPK and Sirt1 activation and stimulates fatty acid oxidation. HepG2 cells were treated with sildenafil (Sild, 1 nM), metformin (Met, 0.1 mM), and leucine (Leu, 0.5 mM) as indicated for 24 hours after induction of lipid accumulation and compared to nontreated cells (control or CTRL high) after lipid accumulation and without lipid accumulation (CTRL low). (a) Sirt1 and (b) AMPK and phospho-AMPK protein expression was measured via Western blotting. Representative blots are shown. Data from repeated experiments are analyzed and presented as mean ± SEM (*n* = 2 to 8). (c) Oxygen consumption rate (OCR) after 200 *μ*M palmitate injection was measured and the area under the curve (AUC) was calculated. Data are represented as mean ± SEM (*n* = 5).

**Figure 2:**
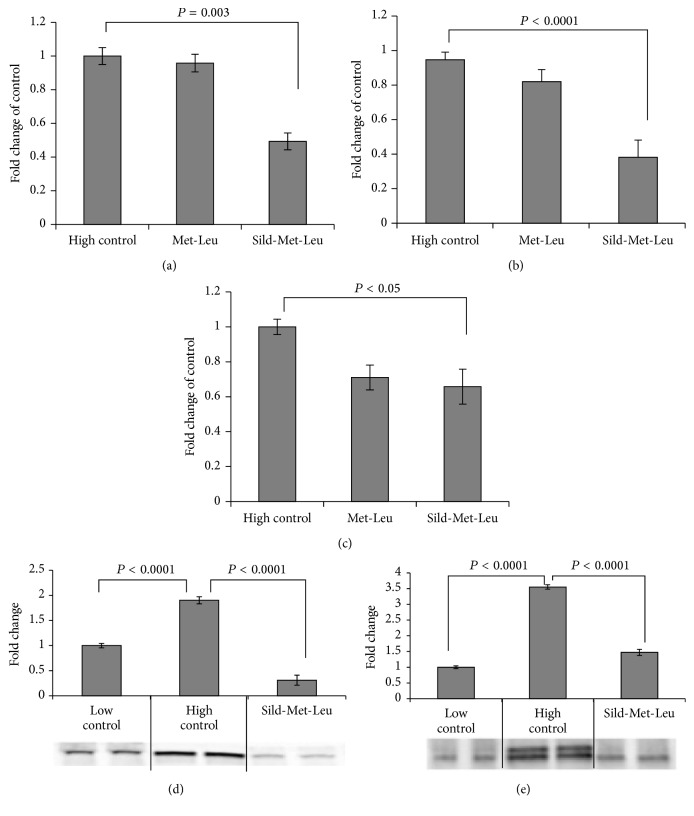
Sild-Met-Leu treatment in hepatocytes decreases lipogenic gene expression. HepG2 cells were treated with sildenafil (Sild, 1 nM), metformin (Met, 0.1 mM), and leucine (Leu, 0.5 mM) as indicated for 24 hours after induction of lipid accumulation and compared to nontreated cells after lipid accumulation (high control) or without lipid accumulation (low control). (a) Fatty acid synthase (FAS), (b) stearoyl-coenzyme A desaturase 1 (SCD1), and (c) acetyl-coenzyme A carboxylase 1 alpha (ACC 1 alpha) gene expression were measured. Data are presented as mean ± SEM (*n* = 8 to 12). ((d) and (e)) Protein expression of FAS and SCD1: quantitative data, presented as mean ± SEM, and representative blots are shown (*n* = 4).

**Figure 3:**
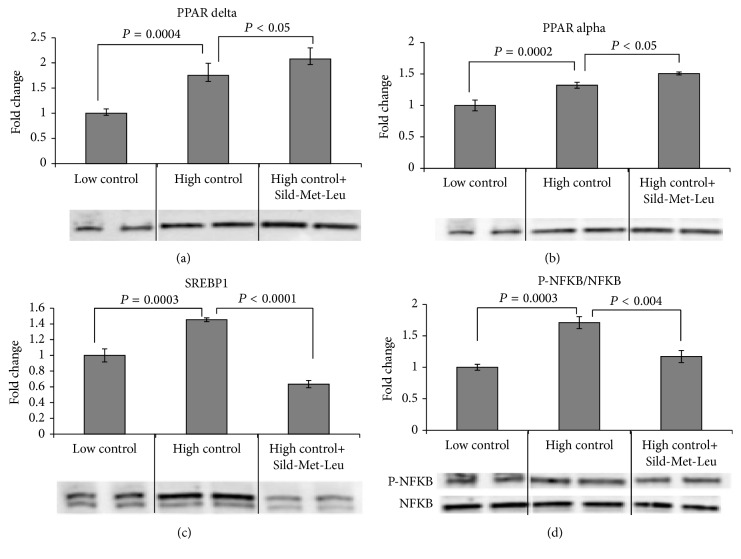
Sild-Met-Leu treatment in hepatocytes increases PPAR alpha and delta and decreases SREBP1 and NF-*κ*B. HepG2 cells were treated with sildenafil (Sild, 1 nM), metformin (Met, 0.1 mM), and leucine (Leu, 0.5 mM) as indicated for 24 hours after induction of lipid accumulation and compared to nontreated cells after lipid accumulation (high control) or without lipid accumulation (low control). Protein expression of (a) PPAR delta, (b) PPAR alpha, (c) sterol regulatory element-binding protein (SREBP) 1, and (d) ratio of phosphorylated to total NF-*κ*B was measured. Quantitative data are presented as mean ± SEM (*n* = 4) and representative blots are shown.

Based on our previous results* in vitro* and* in vivo* showing interacting effects of leucine with either low dose metformin or with PDE5 inhibitors (icariin, sildenafil) on hepatic lipid metabolism, we tested in this study the three-way interaction of leucine, metformin, and sildenafil. As expected, incubation of HepG2 cells with high glucose (25 mM) medium for 48 hours caused significant downregulation of the AMPK/Sirt signaling. This was completely reversed by the three-way combination Sild-Met-Leu while the two-way combinations Met-Leu and Sild-Leu exerted a significant smaller effect (Figures 1(a) and 1(b)). The individual components had no effects. Accordingly, the palmitate-induced oxygen consumption rate in HepG2 cells, measured as a downstream effect of Sirt1/AMPK activation, was significantly increased by the three-way combination. This effect was greater than that exerted by the two-way combinations or by the individual compounds, which had no effect (Figure 1(c)). In addition, gene and protein expressions of lipogenic enzymes (FAS, SCD1 and ACC alpha), which were 2- to 3-fold upregulated after incubation with high glucose medium, were significantly suppressed by Sild-Met-Leu (Figure 2). In addition, the high glucose induced increase of SREBP1, a key transcription factor for lipid synthesis, was reversed by Sild-Met-Leu (Figure 3(c)). In contrast, PPAR alpha and delta, transcription factors regulating fatty acid oxidation, were augmented by Sild-Met-Leu (Figures 3(a) and 3(b)). Moreover, Sild-Met-Leu treatment reduced significantly the ratio of phospho-NF-*κ*B to NF-*κ*B, indicating a reduced inflammatory response (Figure 3(d)).

Next we tested whether these effects could be repeated using a different induction scheme and a different hepatocyte cell line. Induction of lipid accumulation with FFA and LPS had similar effects on Sirt1 and AMPK signaling in HepG2 cells as treatment with high glucose (data not shown). Also, treatment with Sild-Met-Leu increased Sirt1 protein expression in mouse AML-12 hepatocytes and reduced lipogenic protein expression of SREBP1, SCD1, and FAS similar to our observations in HepG2 cells (data not shown). These treatment effects were not caused by significant changes in cell viability (data not shown).

**Figure 4:**
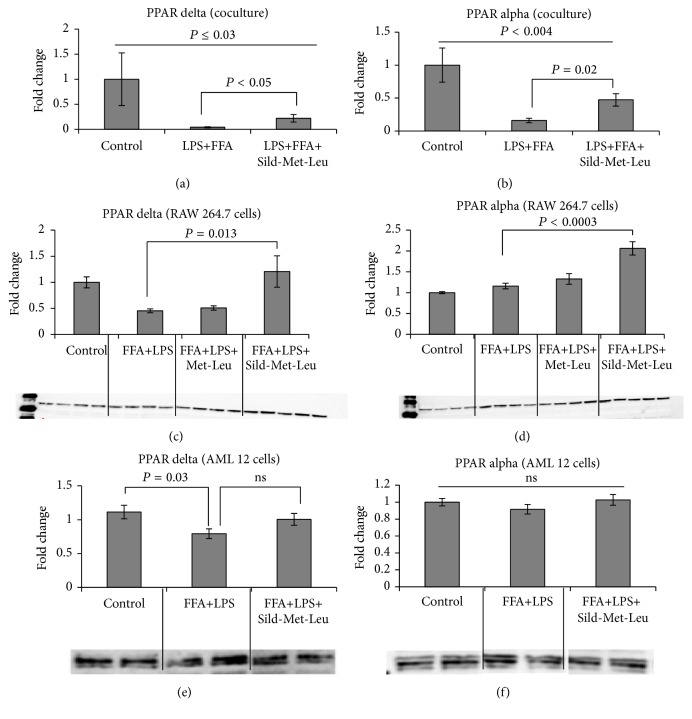
Sild-Met-Leu treatment increases PPAR alpha and delta in mouse hepatocyte-macrophage coculture. Mouse hepatocytes and macrophages, grown individually or in coculture, were treated with sildenafil (1 nM), metformin (0.1 mM), and leucine (0.5 mM) as indicated for 24 hours after induction with free fatty acids (FFA) and LPS. Nontreated cells with induction with FFA and LPS (FFA + LPS) or without (control) were included for comparison. ((a) and (b)) Gene expression of PPAR alpha and delta in hepatocyte-macrophage coculture. Data are represented as mean ± SEM of fold change of control (*n* = 6 to 8). ((c) to (f)) Protein expression of PPAR alpha and delta in RAW 264.7 macrophages and in in AML 12 hepatocytes. Quantitative data are presented as mean ± SEM (*n* = 4 to 5), and representative blots are shown.

**Figure 5:**
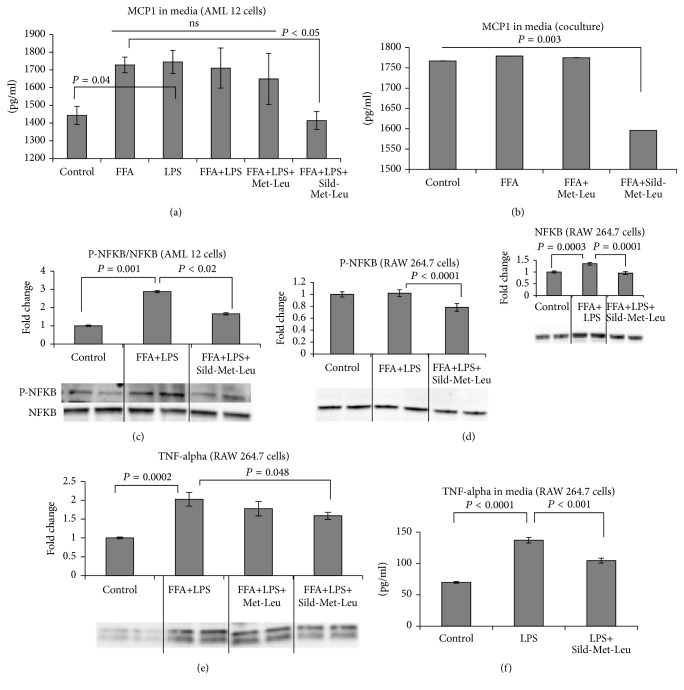
Sild-Met-Leu treatment decreases inflammatory marker in hepatocytes and macrophages. Mouse hepatocytes (AML cells) and macrophages (RAW 264.7 cells), grown individually or in coculture, were treated with sildenafil (1 nM), metformin (0.1 mM), and leucine (0.5 mM) as indicated for 24 hours after induction with free fatty acids (FFA) and/or LPS. Nontreated cells (control) were included for comparison. ((a) and (b)) Monocyte chemotactic protein- (MCP-) 1 secretion in media of AML cells and of hepatocyte-macrophage coculture. Data are presented as mean ± SEM (*n* = 4 to 10). ((c) to (e)) Protein expression of phosphorylated and total NF-*κ*B and TNF-alpha in AML 12 hepatocytes and RAW 264.7 macrophages. Quantitative data are presented as mean ± SEM (*n* = 4), and representative blots are shown. (f) Macrophage tumor necrosis factor- (TNF-) alpha secretion was measured in the media. Data are presented as mean ± SEM (*n* = 5).

Since the activation of macrophages plays an important role in the pathogenesis of NASH, we used mouse hepatocytes (AML 12 cells) and mouse macrophages (RAW 264.7 cells) as an* in vitro* model of NASH. To induce lipid accumulation and an inflammatory response, cells were grown individually or in coculture and stimulated with free fatty acids (oleic/palmitic acid mixture) and/or LPS. Stimulation with LPS and FFA reduced both PPAR alpha and delta in coculture (Figures 4(a) and 4(b)) and treatment with Sild-Met-Leu reversed this effect. Sild-Met-Leu also increased PPAR alpha and delta in macrophages (Figures 4(c) and 4(d)), while there was only a trend (27% increase) for PPAR delta and no effect on PPAR alpha (Figures 4(e) and 4(f)) in AML 12 cells. Secretion of the inflammatory mediator MCP-1 was increased after stimulation of cells with FFA only, LPS only, or the combination FFA and LPS. Sild-Met-Leu completely reversed this effect in AML 12 cells and AML/RAW coculture (Figures 5(a) and 5(b)). Sild-Met-Leu also reduced the ratio of phospho-NF-*κ*B/total NF-*κ*B in AML 12 cells to normal control levels. However, the ratio was not changed in RAW macrophages, since Sild-Met-Leu reduced both, total and phospho-NF-*κ*B (Figures 5(c) and 5(d)). In addition, FFA and LPS induced TNF *α* secretion and protein expression was significantly decreased by Sild-Met-Leu in RAW macrophages (Figures 5(e) and 5(f)).

**Figure 6:**
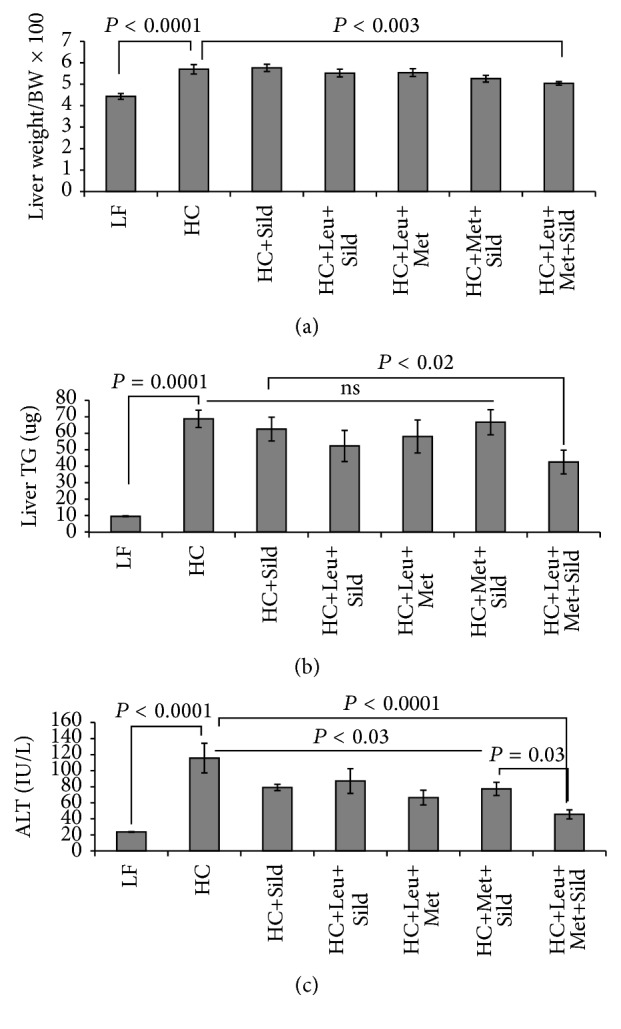
Sild-Met-Leu effects on liver weight, liver triglycerides, and ALT in mice. Mice were fed a low-fat (LF) diet or high-fat atherogenic (HC) diet for 6 weeks and then randomized to the indicated treatment groups for an additional 6 weeks. (a) Liver weight (expressed as ratio to total body weight), (b) liver triglycerides, and (c) alanine aminotransferase (ALT) were determined at the end of the study. Data are expressed as mean ± SEM (*n* = 5 to 8). Sild: sildenafil, Met: metformin, Leu: leucine.

**Figure 7:**
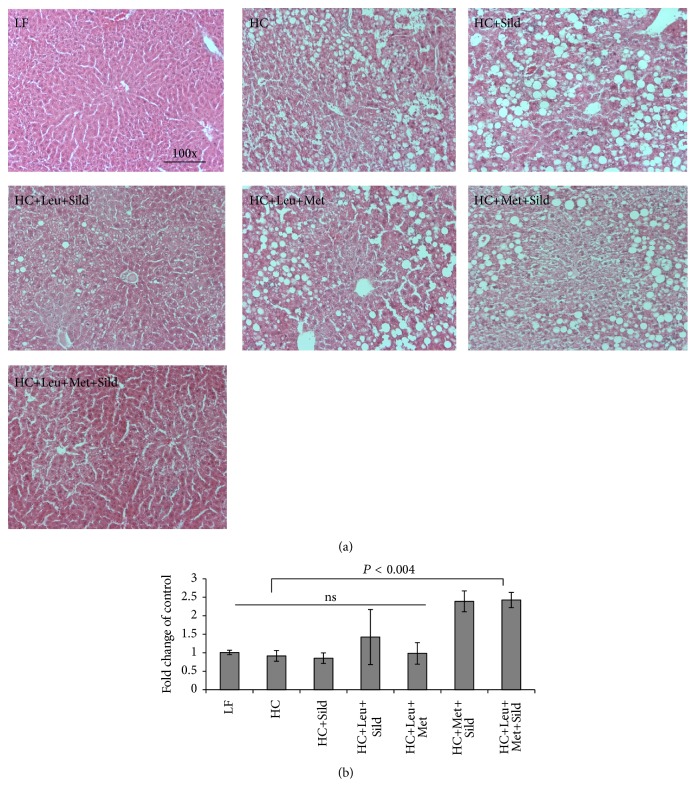
Sild-Met-Leu effects on liver histology in mice. Mice were fed a low-fat (LF) diet or high-fat atherogenic (HC) diet for 6 weeks and then randomized to the indicated treatment groups for an additional 6 weeks. (a) Liver sections were stained with hematoxylin and eosin (H&E) at the end of the study. Representative images for each group are shown. (b) PPAR alpha gene expression in liver extracts was measured and expressed as mean ± SEM of fold change of control (*n* = 6). Sild: sildenafil, Met: metformin, Leu: leucine.

**Figure 8:**
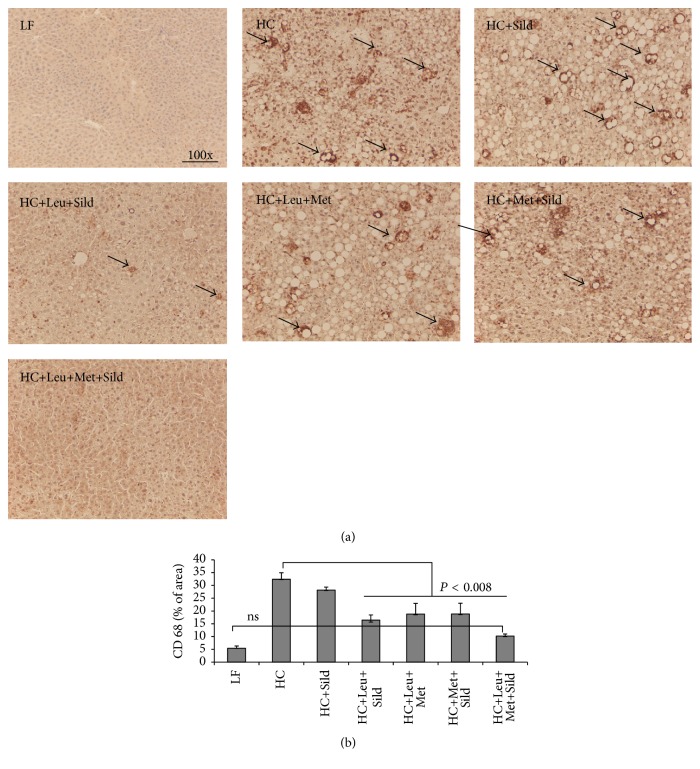
Sild-Met-Leu effects on liver Kupffer cell activation in mice. Mice were fed a low-fat (LF) diet or high-fat atherogenic (HC) diet for 6 weeks and then randomized to the indicated treatment groups for an additional 6 weeks. (a) Liver sections were stained with anti-CD68 antibody at the end of the study (*n* = 2). Representative images for each group are shown. (b) Quantitation of CD68 staining, calculated as % of the examined area. Data are expressed as mean ± SEM (*n* = 2). Sild: sildenafil, Met: metformin, Leu: leucine.

**Figure 9:**
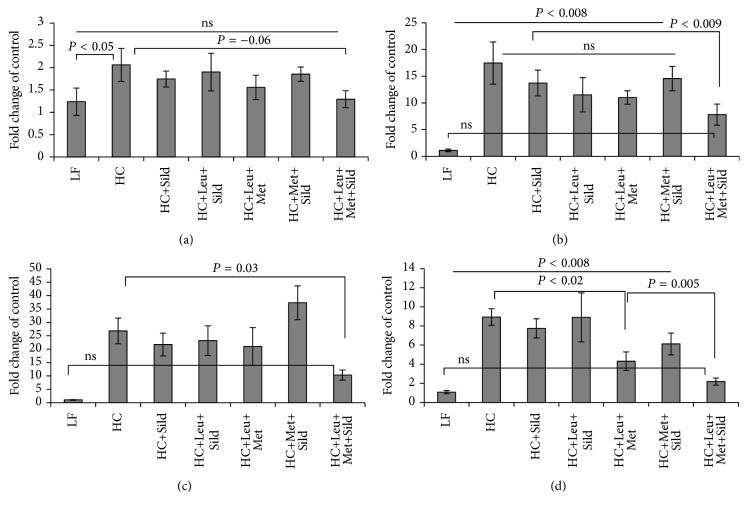
Sild-Met-Leu effects on liver inflammatory marker in mice. Mice were fed a low-fat (LF) diet or high-fat atherogenic (HC) diet for 6 weeks and then randomized to the indicated treatment groups for an additional 6 weeks. Gene expression of (a) interleukin- (IL-) 1 beta, (b) tumor necrosis factor- (TNF-) alpha, (c) monocyte chemotactic protein- (MCP-) 1, and (d) plasminogen activator inhibitor- (PAI-) 1 was measured in liver extracts. Data are expressed as mean ± SEM of fold change of control (*n* = 6). Sild: sildenafil, Met: metformin, Leu: leucine.

**Figure 10:**
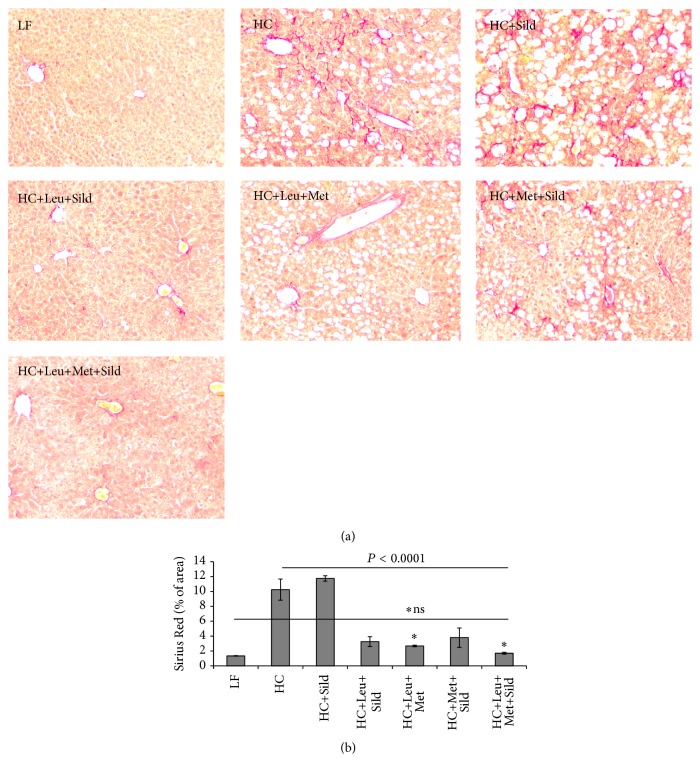
Met-Leu effects on hepatic fibrosis in mice. Mice were fed a low-fat (LF) diet or high-fat atherogenic (HC) diet for 6 weeks and then randomized to the indicated treatment groups for an additional 6 weeks. (a) Liver sections were stained with Picro Sirius Red for collagen at the end of the study (*n* = 3). Representative images for each group are shown. (b) Quantitation of Picro Sirius Red staining, calculated as % of the examined area. Data are expressed as mean ± SEM (*n* = 3). Sild: sildenafil, Met: metformin, and Leu: leucine. *∗* indicates groups which are not significantly different from LF.

**Figure 11:**
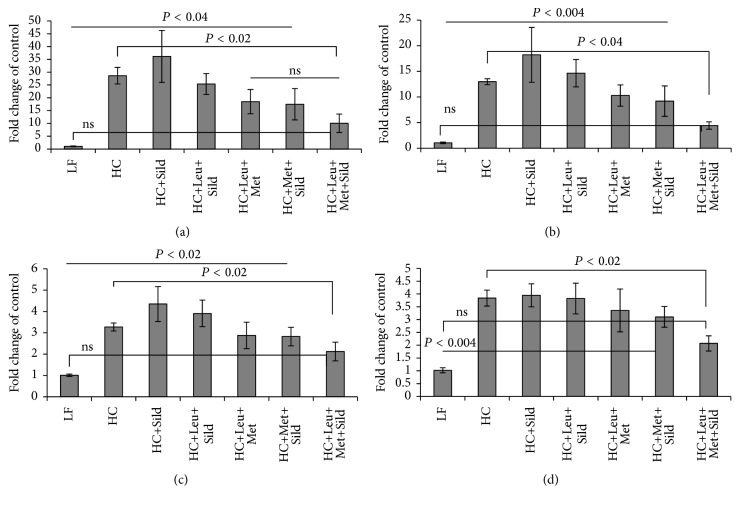
Sild-Met-Leu effects on fibrosis marker in mice. Mice were fed a low-fat (LF) diet or high-fat atherogenic (HC) diet for 6 weeks and then randomized to the indicated treatment groups for an additional 6 weeks. Gene expression of the collagens (a) Col1a1, (b) Col1a2, (c) Col4a1, and (d) transforming growth factor- (TGF-) beta was measured in liver extracts and expressed as mean ± SEM of fold change of control (*n* = 6). Sild: sildenafil, Met: metformin, Leu: leucine.

Based on the* in vitro* data, we assessed the* in vivo* effects of Sild-Met-Leu in comparison with Met-Leu, Met-Sild and Sild-Leu in a NASH mouse model. Feeding of a high-fat atherogenic diet (HC) increased liver weight, liver triglycerides, and ALT levels (sixfold), indicating significant hepatocellular injury, while treatment with the Sild-Met-Leu combination significantly blunted these effects. Although the two-way combinations and sildenafil by itself had some effect on ALT levels, the three-way combination exerted a significantly greater effect in comparison with all other groups (Figure 6). Histology staining confirmed a pronounced increase in lipid droplets and ballooned hepatocytes induced by HC diet compared with low-fat diet control. While the two-way combinations attenuated these effects, the triple combination Sild-Met-Leu substantially reversed the steatohepatitis (Figure 7(a)). Moreover, both Met-Leu and Sild-Met-Leu increased PPAR alpha expression in the liver twofold (Figure 7(b)), consistent with activation of hepatic fatty acid oxidation. To assess the level of inflammation in the liver, sections of liver were stained with CD68. The HC diet caused a sixfold increase of CD68 staining in the liver sections, representing a substantial increase in Kupffer cell activation (Figure 8). All two-way combinations significantly attenuated this effect, while only the three-way combination fully reversed it to levels not significantly different from low-fat fed animals (Figure 8). Consistent with this, inflammatory markers such as IL1 beta, TNF-alpha, MCP-1, and PAI-1 were reduced to normal levels by Sild-Met-Leu, but not by the two-way combinations (Figure 9). Next, we assessed fibrosis in liver sections via Sirius Red staining. The increase in fibrotic changes induced by the HC diet was substantially reversed by Sild-Met-Leu and to a lesser degree by the two-way combinations (Figure 10). In accordance with this, gene expressions of the fibrotic markers Col1a1, Col1a2, Col4a1, and TGF-beta were decreased to normal levels by Sild-Met-Leu but only partly reduced by Met-Leu and Sild-Met (Figure 11).

## 4. Discussion

Our data indicate that the triple combination of leucine, metformin, and sildenafil substantially regresses hepatic steatosis, inflammation, and fibrosis and exerts greater effects than the two-way combination, suggesting that this combination may provide a new therapeutic approach to treat NASH.

The pathophysiology of the development of NASH is thought to be a “multihit process,” where multiple environmental, dietary, and genetic factors interact with others [24]. The accumulation of excess lipids in the liver is considered the first step and the prerequisite for subsequent events, which causes progression from simple steatosis to the severe form of NASH in about 30% of patients with NAFLD. Among the other factors contributing to the progression of NASH, inflammation plays an important role [25]. Chronic injury to hepatocytes or hepatocyte death due to excess free fatty acid influx leads to activation of resident macrophages (Kupffer cells) as well as other infiltrating monocytes and macrophages to release proinflammatory cytokines, including TNF-*α*, IL-1 beta, and IL-6, and profibrogenic factors such as TGF-*β* which in turn results in activation of hepatic stellate cells and fibrosis progression [26, 27].

**Figure 12:**
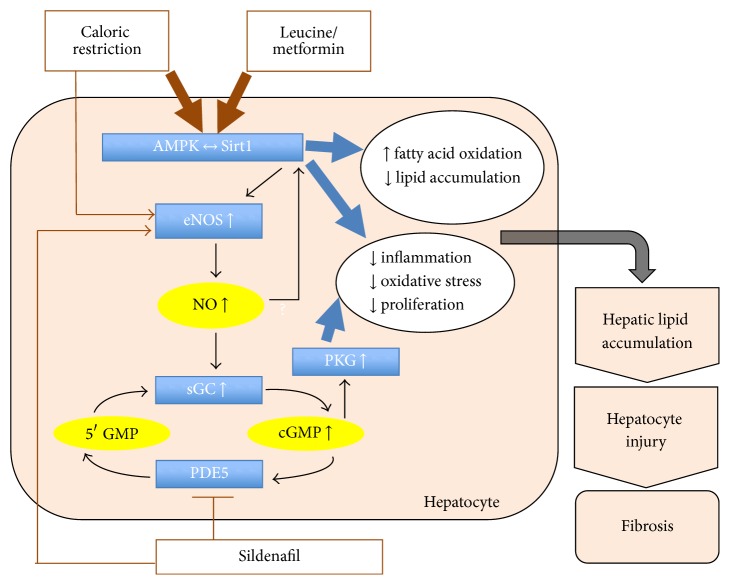
Proposed model of the interaction of leucine, metformin, and sildenafil on the AMPK/Sirt1/eNOS network. AMPK, Sirt1, and eNOS are nutrient sensors responsive to caloric restriction, regulating energy metabolism in an interacting network. In addition, they prevent inflammation and reduce oxidative stress and proliferation, the key factors for the progression of NAFLD to NASH. Leucine and metformin synergistically activate the AMPK/Sirt1 pathway while sildenafil contributes to further stimulation through activation of eNOS. Moreover, sildenafil's inhibition of PDE5 results in concomitant activation of the cGMP-dependent protein kinases (PKGs). These integrated effects result in reduction of hepatic lipid accumulation, hepatic inflammation and injury, and subsequently reduction of fibrosis.

The three-way combination Leu-Met-Sild targets the AMPK-Sirt1-eNOS network, as depicted in Figure 12. AMPK, Sirt1, and eNOS are key regulators of hepatic energy and lipid metabolism, as well as inflammation, oxidative stress, and cell proliferation, the key factors for progression of simple NAFLD to NASH and liver fibrosis [2, 14, 28, 29]. Downregulation of Sirt, AMPK, or eNOS promotes the progression of NASH, while activation of this network has been shown to improve hepatic steatosis and inflammation. For example, eNOS-knockout mice fed a high-fat diet showed more extensive hepatic lipid accumulation and inflammation than wild-type mice [30] and resveratrol treatment, a known Sirt1 and AMPK activator, prevented and reversed lipid accumulation, oxidative stress, and inflammation* in vitro* and* in vivo* [31, 32]. We found the triple combination Sild-Met-Leu to upregulate AMPK and Sirt1 and to increase palmitate-stimulated oxygen consumption and decrease the expression of lipogenic genes such as FAS, ACC, and SCD1 in HepG2 cells. Moreover, treatment with Sild-Met-Leu lowered liver triglycerides and reversed the HFD-induced steatosis in mice, the prerequisite condition for developing NASH. In addition, we show a reduction of inflammatory markers* in vitro* and* in vivo* as well as a normalization of the CD68 staining in liver samples, a marker expressed by monocytes and macrophages. This was also repeated for F4/80 marker in a follow-up mouse study (data not shown), in which F4/80 was reduced by 40% by Sild-Met-Leu feeding to levels not statistically different from the control low-fat fed animals. Therefore, the triple combination also significantly improves inflammation, one of the key factors for driving progression of the disease. In support of this, the HC-induced fibrosis was totally reversed by supplementation with Sild-Met-Leu, indicated by the percentage of Sirius Red positive area in liver.

AMPK and Sirt1 are well-known regulators of hepatic metabolism. However, there is an increasing body of evidence for a role of eNOS and NO/cGMP signaling in the development of hepatic steatosis, inflammation, and progression to fibrosis [14, 30]. The liver is a highly vascularized tissue and eNOS-derived NO from sinusoidal endothelial cells (SEC) regulate vascular resistance, proliferation, and migration, as well as exerting paracrine effects on adjacent stellate cells. As the first cells exposed to portal vein components and bacterially derived lipopolysaccharides (LPS) from the gut, SECs can undergo dramatic phenotype changes and can induce inflammation and stellate cell activation [33]. eNOS is constitutively expressed in SEC and NO plays a crucial role in maintaining physiological phenotypes of SECs and stellate cells [14, 34]. NAFLD is associated with decreased eNOS activation [35]. Moreover, endothelial dysfunction and reduced NO production have been found to precede inflammation and fibrosis in a NAFLD rat model [13]. In contrast, activation of eNOS as well as increased NO production ameliorates the progression of NASH-related hepatic fibrosis [36, 37]. We previously demonstrated the amplifying effects of the Met-Leu combination on AMPK signaling and reduction of hepatic steatosis in DIO-mice [12]. Similarly, leucine with PDE5 inhibitors (sildenafil, icariin) increased fat metabolism and reduced hepatic lipid accumulation in DIO-mice which was associated with increased NO production in addition to AMPK/Sirt1 activation, indicating that the actions of the PDE5 inhibitors converge on this pathway [8, 22]. In this study, we demonstrate that the Sild-Met-Leu combination exerts greater effects on inflammatory and fibrogenic parameters than the Met-Leu or the Sild-Leu combination, suggesting that the stimulation of eNOS/NO/cGMP pathway may contribute to additional effects on the AMPK/Sirt1 signaling.

The peroxisome proliferator-activated receptors- (PPAR-) alpha and delta are transcription factors finely regulating energetic fluxes and metabolic pathways [38]. PPAR-*α* is highly expressed in liver and regulates the rates of fatty acid catabolism and lipogenesis in response to nutritional demands. PPAR-*α* deficient mice develop more severe hepatic steatosis, inflammation, and NASH when fed a HFD compared to wild-type mice [39, 40], while administration of the PPAR-*α* agonists reverses hepatic steatosis and fibrosis [41, 42]. PPAR-*δ* is constitutively expressed and regulates *β*-oxidation in muscle. In the liver, it controls hepatic glucose and lipoprotein metabolism and exerts anti-inflammatory effects [38, 43]. Beneficial effects of PPAR-*δ* agonists on improvement of hepatic steatosis and inflammation have been reported in mouse models of NASH [44]. The three-way combination in this study showed significant upregulation of PPAR-*α* and -*δ in vitro* and of PPAR-*α* in the mouse liver. This may be an indirect treatment effect secondary to AMPK/Sirt1 stimulation, since AMPK and Sirt1 interact with PPAR-*α* and -*δ* [6, 45, 46].

We recently demonstrated the efficacy of the Met-Leu combination and a combination of leucine with the PDE5 inhibitor icariin in reducing hepatic lipid accumulation and inflammation in a HFD-induced NAFLD mouse model [12, 22]. In this study, we used a high-fat atherogenic diet (60% fat, 1.25% cholesterol, and 0.5% cholate) to induce a more severe form of NASH, as this diet induces hepatic insulin resistance, progressive steatosis, inflammation, and fibrosis over 6 to 24 weeks, mimicking the human disease pathology [47]. The animals used in this study developed a significant steatosis with ~7-fold increase in liver triglycerides, hepatic inflammation, and fibrosis within the 12 weeks of study, which is comparable to other studies using this form of diet to induce NASH [48–50].

There are some limitations to this study. We used different forms of induction for the lipid accumulation in HepG2 (high glucose) and AML cells (high concentration of FFA), which limits the ability to compare results between these cell lines. Moreover, AML and RAW cells were stimulated with FFA, LPS, or a combination of both in different experiments, although we show in Figure 3(a) that there was a comparable effect. Finally, not all* in vitro* parameters were measured* in vivo* due to limited tissue availability and since we had demonstrated AMPK/Sirt1 activation already previously for the two-way combinations in mice studies [12, 22].

In summary, we demonstrate the beneficial effects of the three-way combination Sild-Met-Leu on the reversal of hepatic steatosis, inflammation, and fibrosis in a NASH mouse model and that all the three components are necessary for maximal effect. These effects are mediated by targeting the AMPK/Sirt1/eNOS network from multiple sites, each contributing a modest effect to the overall outcome, as summarized in Figure 12. This approach allows a substantial dose reduction of each individual compound to a concentration, which has little or no independent effect on the measured outcomes. Therefore, the risk of associated adverse effects of the individual compounds will be diminished. Based on the pivotal role of the AMPK/Sirt1/eNOS network in hepatic metabolism and the promising results of this animal study, the Sild-Met-Leu combination provides a new therapeutic approach to treat NAFLD and NASH.

## Abbreviations

eNOS: Endothelial nitric oxide synthase
NO: Nitric oxide
5′GMP: 5′Guanosine monophosphate
cGMP: Cyclic guanosine monophosphate
sGC: Soluble guanylate cyclase
PKGs: cGMP-dependent protein kinases
PDE5: Phosphodiesterase 5.
